# Validation of Artificial Intelligence Computer-Aided Detection on Gastric Neoplasm in Upper Gastrointestinal Endoscopy

**DOI:** 10.3390/diagnostics14232706

**Published:** 2024-11-30

**Authors:** Hannah Lee, Jun-Won Chung, Sung-Cheol Yun, Sung Woo Jung, Yeong Jun Yoon, Ji Hee Kim, Boram Cha, Mohd Azzam Kayasseh, Kyoung Oh Kim

**Affiliations:** 1 Division of Gastroenterology, Department of Internal Medicine, Gachon University, Gil Medical Center, Incheon 21565, Republic of Korea; everagape@gmail.com (H.L.); junwonchung@daum.net (J.-W.C.); 2 Division of Biostatistics, Center for Medical Research and Information, University of Ulsan College of Medicine, Seoul 05505, Republic of Korea; ysch97@amc.seoul.kr; 3 Division of Gastroenterology, Department of Internal Medicine, Korea University College of Medicine, Ansan 15355, Republic of Korea; sungwoojung@korea.ac.kr; 4 CAIMI Co., Ltd., Incheon 22004, Republic of Korea; yjyoon@caimi.co.kr (Y.J.Y.); gilgi-jhkim@naver.com (J.H.K.); 5 Division of Gastroenterology, Department of Internal Medicine, Inha University Hospital, Inha University School of Medicine, Incheon 22332, Republic of Korea; chaboram@hanmail.net; 6 Division of Gastroenterology, Dr. Sulaiman AI Habib Medical Group, Dubai Healthcare City, Dubai 51431, United Arab Emirates; drmakdxb@gmail.com

**Keywords:** artificial intelligence, computer-aided detection (CADe) algorithm, gastric neoplasm

## Abstract

Background/Objectives: Gastric cancer ranks fifth for incidence and fourth in the leading causes of mortality worldwide. In this study, we aimed to validate previously developed artificial intelligence (AI) computer-aided detection (CADe) algorithm, called ALPHAON^®^ in detecting gastric neoplasm. Methods: We used the retrospective data of 500 still images, including 5 benign gastric ulcers, 95 with gastric cancer, and 400 normal images. Thereby we validated the CADe algorithm measuring accuracy, sensitivity, and specificity with the result of receiver operating characteristic curves (ROC) and area under curve (AUC) in addition to comparing the diagnostic performance status of four expert endoscopists, four trainees, and four beginners from two university-affiliated hospitals with CADe algorithm. After a washing-out period of over 2 weeks, endoscopists performed gastric detection on the same dataset of the 500 endoscopic images again marked by ALPHAON^®^. Results: The CADe algorithm presented high validity in detecting gastric neoplasm with accuracy (0.88, 95% CI: 0.85 to 0.91), sensitivity (0.93, 95% CI: 0.88 to 0.98), specificity (0.87, 95% CI: 0.84 to 0.90), and AUC (0.962). After a washing-out period of over 2 weeks, overall validity improved in the trainee and beginner groups with the assistance of ALPHAON^®^. Significant improvement was present, especially in the beginner group (accuracy 0.94 (0.93 to 0.96) *p* < 0.001, sensitivity 0.87 (0.82 to 0.92) *p* < 0.001, specificity 0.96 (0.95 to 0.97) *p* < 0.001). Conclusions: The high validation performance state of the CADe algorithm system was verified. Also, ALPHAON^®^ has demonstrated its potential to serve as an endoscopic educator for beginners improving and making progress in sensitivity and specificity.

## 1. Introduction

Gastric cancer ranks fifth for incidence and fourth in the leading causes of mortality worldwide [1]. According to up-to-date global cancer statistics, 968,350 people worldwide are newly diagnosed with gastric cancer each year with 659,853 deaths every year [1]. Referring to the worldwide distribution of gastric cancer, East Asia and Eastern Europe have the highest rates of incidence [1,2]. In addition, the expected incidence of gastric cancer in the next decade is high taking consideration into the increasing aged population [3]. Also, in the aspect of comparing the significant difference in prognosis and 5 year survival rate between early and advanced gastric cancer, specifically, a 90% 5 year survival rate versus nearly 20%, detecting gastric cancer in the early stage is important for favorable disease outcomes [4,5,6]. Early detection with diagnosis in the early stage and screening patients in high-risk states is evaluated and regarded as an effective strategy for reducing medical and clinical burden by prompt intervention after early detection with favorable disease outcomes [7,8,9,10,11]. Gastroscopy is related to a further 67% reduction of gastric cancer compared with radiological evaluation, and endoscopic management significantly decreased its mortality [12,13,14,15,16]. Therefore the importance of diagnosing early gastric neoplasm is an upcoming issue in the endoscopic clinical field regarding mortality and prognosis [4,5,6]. Detecting and making the diagnosis in the earlier disease stage is not a simple process owing to its clinical diminutive symptom and endoscopic subtle change from background gastric mucosa and generalized inflammation [17,18,19]. Development of endoscopic optical devices which includes narrow-band imaging (NBI) and magnification endoscopy in combination with white light endoscopy (WLE), has been introduced in the endoscopic field to assist and improve its diagnostic capability [20,21]. However, depending on the human eye and subjective decision, missing rate and interval cancer are inevitable in endoscopic diagnosis [22,23]. The presence of skill variations and range among endoscopists in detecting dysplastic lesions leads to different disease outcomes [24,25]. The missing rate of early gastric cancer (EGC) in endoscopic screening tests reaches as high as 10% within a 3 year period from the previous endoscopic examination [22,23]. In accordance with gastric lesions, previous prior studies have demonstrated current endoscopic imaging modalities including narrow-band imaging as well as advanced endoscopic tools such as magnification endoscopy lack in detecting and localizing dysplastic gastric mucosal lesions including early gastric cancer more precisely in the aspect of sensitivity and specificity [26,27,28,29].

In recent years, artificial intelligence (AI) systems have been introduced and presented capability in diverse medical fields to assist endoscopists in making clinical decisions [30,31,32,33,34,35,36,37,38]. Since artificial intelligence (AI) rapidly engages in the field of medicine continuously, transparent development of clinical applications is undeniable [39]. Considerably dependent on image study of endoscopic and radiologic evaluation, the field of gastroenterology has been a highly applicable area of AI technology. Various areas, including the detection of precancerous or cancerous lesions and improvement of misdiagnosis, have given special interest [40,41,42]. Identifying and localizing premalignant and early gastric malignancy is essential, taking worldly high cancer-related mortality into consideration. Deep learning mainly requires tasks of computer-aided detection (CADe) and computer-aided diagnosis (CADx) which each function as real-time detection and characterization. CADe usually marks and presents both detection and localization of the expected lesion, whereas CADx results in diagnosis with differentiation.

Though limitations of datasets composing and training AI systems exist, it has demonstrated improvement and potential to detect and diagnose endoscopic lesions allowing and fulfilling the goal of earlier detection and management of precancerous lesions [43,44,45,46,47,48].

In this study, we aimed to validate previously developed artificial intelligence (AI) computer-aided detection (CADe) algorithm system in our latest work, called ALPHAON^®,^ and to compare outcomes with previous studies presenting AI outperforming and assisting endoscopists of diverse levels of expertise in detecting gastric neoplasm [49,50].

## 2. Materials and Methods

### 2.1. Study Design and Participants

**Figure 1:**
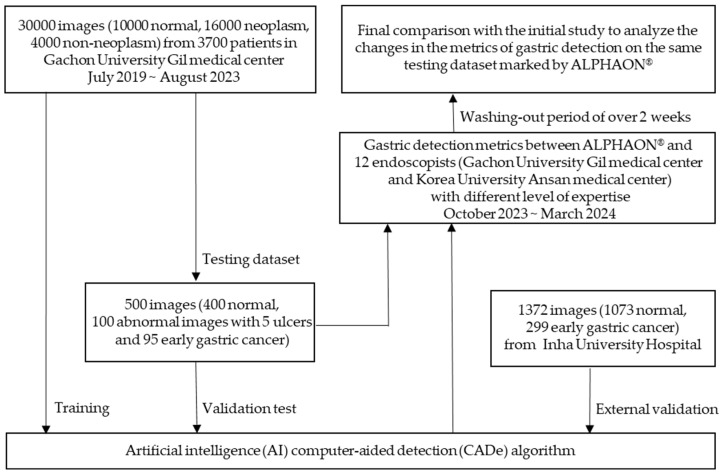
Workflow for development and validation of the artificial intelligence (AI) computer-aided detection (CADe) algorithm.

This study is a retrospective, multi-center, controlled diagnostic study performed in two university-affiliated medical centers (e.g., Gachon University Gil Medical Center and Korea University Ansan Medical Center) in the Republic of Korea from 25 October 2023 to 29 March 2024. The overall workflow for the development and validation of the artificial intelligence (AI) computer-aided detection (CADe) algorithm is presented in Figure 1.

To evaluate and validate the performance state of ALPHAON^®^ (CAIMI Co., Ltd., Incheon, Republic of Korea) and generalize its applicability, retrospectively obtained 500 stomach endoscopic images containing both 400 normal images and 100 abnormal (e.g., 5 ulcers, and 95 early gastric cancer) images have been validated by endoscopists of diverse levels of expertise from two university-affiliated medical centers.

The study was designed to assess the performances of ALPHAON^®^ and endoscopists using two different methods:

(1) Compare gastric detection metrics (i.e., accuracy, sensitivity, specificity) between ALPHAON^®^ and endoscopists across datasets of 500 stomach endoscopic images containing both 400 normal images and 100 abnormal (e.g., 5 ulcers, and 95 early gastric cancer) images.
(2) After a washing-out period of over 2 weeks, endoscopists performed gastric detection on the same dataset of the 500 endoscopic images again marked by ALPHAON^®^. Subsequently, endoscopists utilize ALPHAON^®^’s detection results to determine the final diagnosis. Final comparison of these results with the initial study was performed to analyze the changes in the metrics.

### 2.2. Data Preparation and Image Quality Control

A total of 30,000 endoscopic images, including 10,000 normal images, 16,000 of neoplasm, and 4000 of non-neoplasm were retrospectively collected from 3700 patients who had undergone gastroendoscopic exams during July 2019 and August 2023 in Gachon University Gil Medical Center. Abnormal findings and site of neoplasm and non-neoplasmic endoscopic images were pooled to form region of interest (ROI) and utilized for training and validation tests. With obtained endoscopic data sets, 10-fold cross validation was processed to prevent overfitting and the performance of detection sensitivity on average was 85% and the error rate per image was 0.04. All endoscopic images were taken and captured in high-resolution quality using different Olympus endoscopes (GIF-H260, GIF-H260Z, GIFHQ290, GIF-H290Z; Olympus Medical Systems, Tokyo, Japan) with video systems (EVIS LUCERA CV260/CLV260SL, EVIS LUCERA ELITE CV290/CLV290SL, Olympus Medical Systems).

In the format of a jpeg, endoscopic images of upper gastrointestinal endoscopy were stored in the database of Gachon University Gil Medical Center. Only white light endoscopic images had eligibility whereas images with staining dye and narrow-band imaging were not included. In addition, images with poor background and quality caused by mucus, halation, blurs, defocusing, less insufflation of air, bubbles, sliding, fuzzy, and bleeding were also excluded. Well-experienced endoscopists from Gachon University Gil Medical Center with more than 5 years of clinical endoscopic experience and had performed over 5000 endoscopic exams were under evaluation on the quality of all the images. Neoplasmic lesions of the upper gastrointestinal study were all labeled manually. Marking was done at the border of each neoplasmic lesion carefully by endoscopists. Any of the endoscopic images that were mismatched with the pathological diagnosis were removed. Experienced endoscopists from Gachon University Gil Medical Center performed quality control, labeling, and delineation. Endoscopists in the same group performed labeling and delineation with cooperation but regarding delineation, one endoscopist processed delineation under the supervision of the other endoscopist. The selection, labeling, and delineation steps were considered completed when the two endoscopists reached the same conclusion.

### 2.3. Development and Training of the Artificial Intelligence (AI) Computer-Aided Detection (CADe) Algorithm

The images from Gachon University Gil Medical Center were assigned to the training and testing datasets for developing ALPHAON^®^. The developed artificial intelligence (AI) computer-aided detection (CADe) algorithm was based on the concept of EfficientNet. Having the advantage and strength of scaling efficiency and high accuracy with limited processing power, EfficientNet has been applied for ALPHAON^®^ with the suitability of real-time usage and practice [51]. The gastric cancer detection method proposed in this study has three steps: image pre-processing, image classification, and gastric lesion detection. We processed 10-fold cross validation to prevent overfitting of the deep learning model. In image pre-processing, retrospectively obtained endoscopic images were reconstructed to have an efficient training time. The classification model with EfficientNet structure was applied to sort out gastric lesion images among endoscopic images to reduce false positives followed by a detection model for gastric lesion detection.

As commonly used other algorithms, DenseNet has the strength of feature reuse connecting each layer to every other layer in a feed-forward manner and also it has the advantage of achieving high performance with fewer parameters [52]. However, it has the limitation of high memory usage during training and slower inference speed owing to the high number of connections [52].

ResNet structure, another widely introduced algorithm, has the power of having residual connections with the over-coming problem of vanishing gradients, though it has the limitation of having demanding computational cost and higher parameter count [53].

Considering its strength of high efficiency in image tasking especially in resource-constrained environments and suitability of real-time application in the practical endoscopic field, EfficientNet has been applied for the development of artificial intelligence (AI) computer-aided detection (CADe) algorithm, ALPHAON^®^.

### 2.4. Validation and Testing of the ALPHAON^®^ and Comparison with Endoscopists

The testing images included 500 images of 100 patients from Gachon University Gil Medical Center between July 2019 and August 2023. Endoscopic images were prepared in high-resolution quality using different endoscopes (GIF-HQ290, GIF-H260, Olympus Medical Systems, Tokyo, Japan). The testing dataset included 100 abnormal images composed of 5 ulcers and 95 early gastric cancer images and 400 normal images to compare the performance of the ALPHAON^®^ and endoscopists. The final pathologic diagnosis of early gastric cancer was confirmed by two board-certified pathologists using haematoxylin- and eosin-stained tissue slides based on the WHO Classification of Tumors.

A total of 12 endoscopists composed of 4 endoscopists from Gachon University Gil Medical Center and 8 endoscopists from Korea University Ansan Medical Center participated as investigators in the validation of ALPHAON^®^. Endoscopists from the two institutions were assigned to three groups based on their level of expertise. By levels and endoscopic clinical expertise, four endoscopists with advanced expertise of more than 15 years of clinical endoscopic experience, four with an intermediate level of experience, and five at the early stage of their practice enrolled in the study.

External validation tests were performed for approval on the generalizability of the developed artificial intelligence (AI) computer-aided detection (CADe) algorithm, ALPHAON^®^ with 1372 endoscopic images (1073 normal, 299 early gastric cancer) from 191 patients (108 normal, 83 early gastric cancer) in Inha University Hospital.

### 2.5. Statistical Analysis

The study evaluated the performance of a model trained for real-time lesion detection during endoscopy, using data from 500 patients. It utilized common performance metrics employed in object detection models, including the area under the ROC curve (AUC) score, accuracy, sensitivity, and specificity. A statistical confidence interval was computed to estimate the probable range of the true parameter. Logistic regression via generalized estimating equations (GEE) was applied to evaluate the result of the initial validation outcome and washing-out period of over 2 weeks later. The statistical method of logistic regression was applied for the prediction of probability on a linear combination of initial validation and outcome and washing-out period in the sense of image classification.

The diagnostic validation of ALPHAON^®^ was accessed by evaluating accuracy, sensitivity, and specificity as primary outcomes of the study by calculating the 95% CIs using the ClopperPearson method. The receiver operating characteristic (ROC) curve was utilized to demonstrate the diagnostic ability of ALPHAON^®^’s deep learning algorithm in detecting and differentiating patients with upper gastrointestinal neoplastic lesions from controls. ROC curves were formed by plotting the proportion of true positive cases (sensitivity) against the proportion of false positive cases (1 − specificity), by varying the predictive probability threshold for evaluation of ALPHAON^®^’s diagnostic validation. A better diagnostic performance was indicated with a larger area under the ROC curve (AUC). All statistical tests were two sided with a significance level of 0.05. Statistical analysis was performed using SPSS (version 26.0; IBM Inc., Armonk, NY, USA) or R software (version 3.6.3).

Accuracy is defined as the deep learning algorithm system identifying a predicted box containing a neoplastic lesion when its confidence value output by the ALPHAON^®^ is bigger than a given cut-off value. In this study, the cut-off value is defined as the value for which the point on the Receiver operating characteristic (ROC) curve has the minimum distance to the upper left corner (where sensitivity = 1 and specificity = 1).

The objective of this study was to demonstrate the superior performance of ALPHAON^®^ in detecting gastric lesions including ulcers and early gastric cancer when compared to endoscopists of diverse levels of expertise; endpoints of the study are listed and categorized below:

Co-Primary Endpoints
λ Accuracy
Formula: (True negative + True positive)/(True negative + False positive + False negative + True positive)
Accuracy = True predictions/Total number of cases
λ Sensitivity
Formula: True positive/(True positive + False negative)
Sensitivity = True positive/Total number of positive cases
λ Specificity
Formula: True negative/(True negative + False positive)
Specificity = True negative/Total number of negative cases
Secondary Endpoints
λ Threshold value
λ AUC (Area Under the ROC Curve) sensitivity
λ AUC specificity.

Higher sensitivity and specificity signify greater diagnostic accuracy. This implies that as TPR approaches 1 and FPR approaches 0, the diagnosis becomes more accurate. For a quantitative comparison of the most efficient threshold value, the AUC is essential. Computing the area under the ROC curve and selecting the threshold value that yields the highest value is the most efficient approach.

### 2.6. Ethics

The study design was reviewed and approved by the Medical Ethics Committee of Gachon University Medical Center (GBIRB2023-167). Retrospectively prepared endoscopic images in the database of each medical center did not require informed consent from participating patients.

## 3. Results

### 3.1. Performance of the ALPHAON^®^

**Figure 2:**
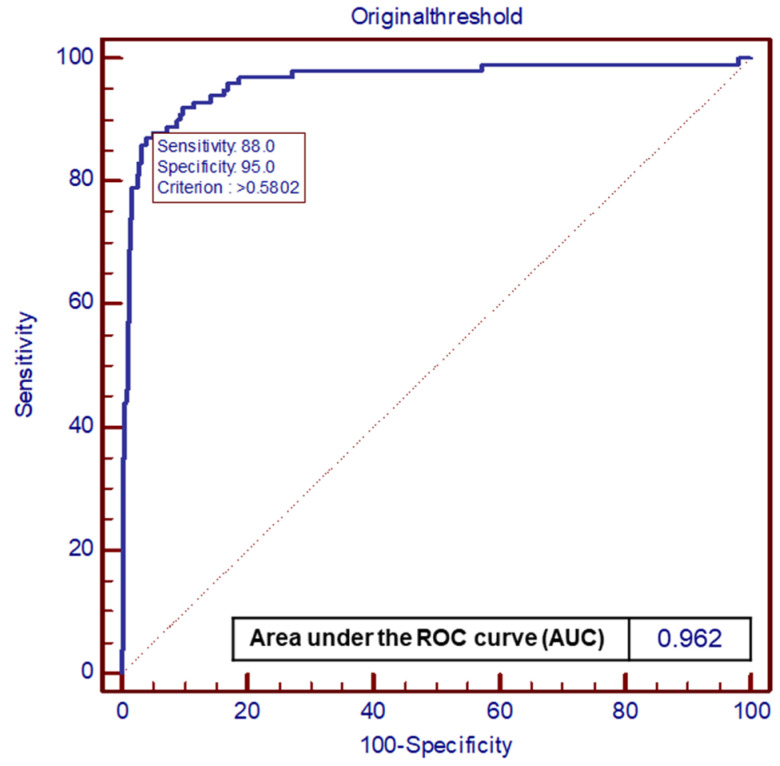
Illustration of ALPHAON^®^’s capability on detecting upper gastrointestinal neoplasm by receiver operating characteristic curve.

**Figure 3:**
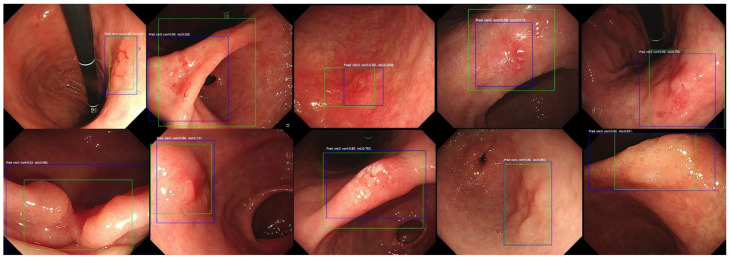
Ten different early gastric cancer endoscopic images of external validation datasets from Inha University Hospital marked with green bounding box for ground truth and blue bounding box for AI detection. IoU has been taken under evaluation on the overlap area between predicted bounding box (blue) and ground truth bounding box (green).

The CADe algorithm presented high validity in detecting gastric neoplasm with accuracy (0.88, 95% CI: 0.85 to 0.91), sensitivity (0.93, 95% CI: 0.88 to 0.98), specificity (0.87, 95% CI: 0.84 to 0.90) (Table 1), and area under the ROC Curve (0.962, criterion > 0.580, sensitivity: 0.88, specificity: 0.95) (Figure 2). These values are exclusively derived from the dataset comprising 500 patients in the study, and consequently, may not accurately represent the entire population. Evaluating detecting status according to endoscopist’s expertise depending on years of endoscopic experience with ALPHAON^®^, there were no significant differences between AI and expert (0.97, 95% CI: 0.94 to 0.99, *p* = 0.170) in sensitivity apart from trainee (0.85, 95% CI: 0.81 to 0.89, *p* = 0.003) and beginner (0.73, 95% CI: 0.67 to 0.79, *p* < 0.001). External validation test from Inha University Hospital has presented a sensitivity of 0.77, specificity of 0.96, accuracy of 0.92 with a threshold of 0.28, and IoU 0.30. Endoscopic images with IoU visualization of external datasets are presented in Figure 3.

### 3.2. Comparison Between the ALPHAON^®^ and Endoscopists

In the first study, a comparison between ALPHAON^®^ and endoscopic participants was evaluated. Endoscopists were categorized into four groups: an expert group with endoscopic specialists with 15 years of clinical endoscopic experience, a trainee group with fellows with an intermediate level of endoscopic experience, and a beginner group with residents in the early stage of their practice.

**Figure 4:**
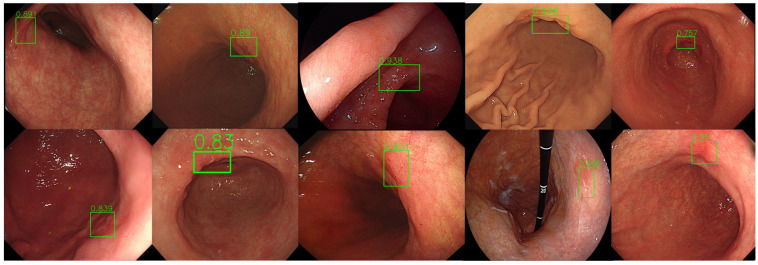
Ten different early gastric cancer endoscopic images of test dataset from Gachon University Gil Medical Center with AI-detected green bounding boxes marked at early gastric cancer lesion by ALPHAON^®^. Expected probability of lesion detection by developed CADe algorithm is marked with green bounding boxes at gastric cancer lesion.

**Figure 5:**
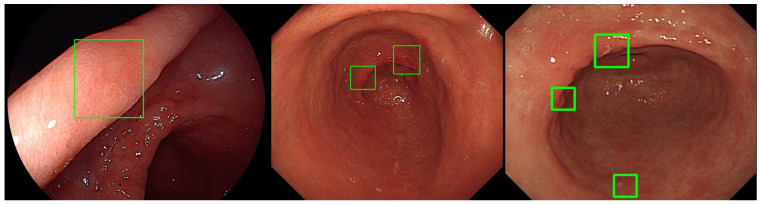
Early gastric cancer endoscopic images of test dataset from Gachon University Gil Medical Center mentioned in Figure 4 with bounding boxes marked by beginners at simple erosions missing detection of cancerous lesions.

**Figure 6:**
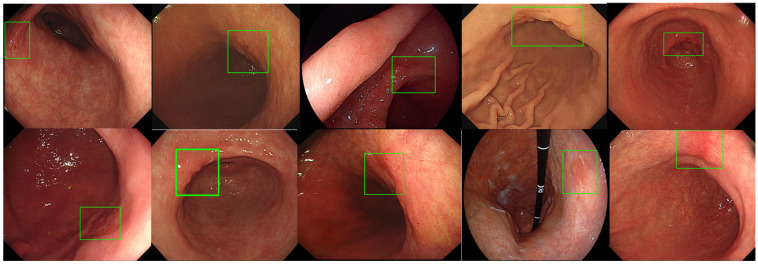
Early gastric cancer endoscopic images of test dataset from Gachon University Gil Medical Center mentioned in Figure 4 with bounding boxes marked at early gastric cancer lesion by expert endoscopists presenting correlated outcome with AI-detected bounding boxes in Figure 4.

Endoscopic images of normal stomach and abnormal images including early gastric cancer and benign ulcers were presented to endoscopic participants of different expertise and AI. Bounding boxes at suspected gastric lesions of abnormality are requested to be marked by endoscopist participants and ALPHAON^®^ for evaluation and comparison of their lesion-detecting validity. Just as presented in Figure 4. Ten different endoscopic images of early gastric cancer were tested and evaluated under ALPHAON^®^ detection. All ten endoscopic images present AI-detected bounding boxes marked at early gastric cancer lesions by ALPHAON^®^. Though the same endoscopic images of normal stomach and abnormal images including early gastric cancer and benign ulcers were presented to endoscopic participants of different expertise and AI, bounding boxes are not marked in accordance with each other group. Figure 5 shows the detection results with the beginner group of residents. With the same early gastric cancer endoscopic images mentioned in Figure 4, bounding boxes were marked at simple erosions by endoscopic beginners, which presents missing cancerous lesion detection. Figure 6 demonstrates the same endoscopic images of early gastric cancer mentioned in Figure 4 with bounding boxes marked at suspected early gastric cancer lesions by expert endoscopists presenting correlated outcomes with AI-detected bounding boxes in Figure 4. Comparing the results of the presented endoscopic figures with bounding boxes marked by endoscopists with different endoscopic expertise and AI, taking into consideration the role of AI as educator and assistant becomes more essential. As the figure shows according to the result between AI and experts compared with endoscopic beginners, utilizing the assistance of AI for endoscopists in the beginning stage is expected to be of great importance.

The sensitivity of ALPHAON^®^ (0.93, 95% CI: 0.88 to 0.98) statistically surpassed that of beginner (0.73, 95% CI: 0.67 to 0.79, *p* < 0.001) and trainee (0.85, 95% CI: 0.81 to 0.89, *p* = 0.003) groups. However, the accuracy (0.88, 95% CI: 0.85 to 0.91), and specificity (0.87, 95% CI: 0.84 to 0.90) of ALPHAON^®^ did not demonstrate superiority over expert (0.99, 95% CI: 0.98 to 0.99, *p* < 0.001, 0.99, 95% CI: 0.99 to 1.00, *p* < 0.001) and trainee (0.96, 95% CI: 0.95 to 0.97, *p* < 0.001, 0.99, 95% CI: 0.98 to 0.99, *p* < 0.001). ALPHAON^®^’s lesion-detecting performance of accuracy and specificity appeared relatively inferior to that of endoscopists, although both achieved results around 0.9.

In the second study, the presence and advancement in endoscopic lesion detection and diagnostic capability of endoscopists with diverse expertise after applying the assistance of ALPHAON^®^ were taken into evaluation. The overall accuracy, sensitivity, and specificity of expert, trainee, and beginner were statistically improved with the assistance of ALPHAON^®^ (Table 1). After a washing-out period of over 2 weeks, validity improved in trainee (accuracy 0.97 (0.96 to 0.98) *p* = 0.021, sensitivity 0.88 (0.83 to 0.92) *p* = 0.168, specificity 0.99 (0.99 to 1.00) *p* = 0.031) and beginner groups (accuracy 0.94 (0.93 to 0.96) *p* < 0.001, sensitivity 0.87 (0.82 to 0.92) *p* < 0.001, specificity 0.96 (0.95 to 0.97) *p* < 0.001) with assistance of ALPHAON^®^ and significant improvement was present especially in beginner group. This assisting-performing status of ALPHAON^®^ improving and making the advancement of the outcome, especially for beginner endoscopists significantly demonstrates AI’s role as an educator.

On the contrary, artificial intelligence assistance resulted in slightly inferior outcomes for the expert group, although the difference was not significant enough to raise concerns (accuracy 0.97 (0.97 to 0.98) *p* = 0.008, sensitivity 0.91 (0.87 to 0.95) *p* = 0.003, specificity 0.99 (0.99 to 1.00) *p* = 0.590).

## 4. Discussion

In this study, we used ALPHAON^®^, artificial intelligence (AI) computer-aided detection (CADe) algorithm to evaluate its validity in detecting gastric neoplasm with metrics of accuracy, sensitivity, and specificity in addition to compare diagnostic capability with human endoscopists by levels of clinical endoscopic experience. Also incorporating a second step study with two weeks of washing-out period utilizing the result of ALPHAON^®^’s detection outcome, we evaluated efficacy and role as guidance in lesion detection of ALPHAON^®^ in gastrointestinal medical field according to different endoscopic expertise.

ALPHAON^®^ has presented sensitivity 0.93 (88.0–98.0%) comparable to that of expert endoscopists and has shown a sensitivity of 0.93 (88.0–98.0%), specificity of 0.87 (84.0%, 90.0%), and AUC value of 0.962 (0.941–0.977) in detecting upper gastrointestinal neoplasm. Specifically, in the second study, the sensitivity of beginner (0.87 (0.82 to 0.92) *p* < 0.001) and trainee (0.88 (0.83 to 0.92) *p* = 0.168) endoscopists presented improvement and only beginner endoscopists showed significant change. Concerning accuracy, beginner (0.94 (0.93 to 0.96) *p* < 0.001) and trainee (0.97 (0.96 to 0.98) *p* = 0.021) endoscopists demonstrated better advanced outcome with AI and a significant result was shown in the beginner endoscopist group.

To our knowledge, this is the first study to not only validate computer-aided detection (CADe) algorithm but also demonstrate clinical beneficial outcome of utilizing AI model in practical clinical field emphasize AI’s role of assisting endoscopists in detecting gastric neoplasm.

Validation of ALPHAON^®^ detection gastric neoplasm in our study is compatible with previous studies examining gastric malignancy or premalignant conditions presenting accuracy in range of 86.5–98.7%, sensitivity of 80.0–96.7%, and specificity of 89.2–100% [43,45,54,55].

It is also compatible with recent multicenter diagnostic studies presenting relevant outcome of expert endoscopist and AI performing as potential endoscopic assistant [45,46], specifically, a multicenter case-control diagnostic study outcome demonstrating diagnostic accuracy of 0.955 (95% CI 0.952–0.957) and sensitivity of 0.942 (95% CI 0.924–0.957), which has presented relevant outcome of experts [45]. Also, another multicenter retrospective diagnostic study has presented comparable results regarding accuracy (85.1–91.2%) and sensitivity (85.9–95.5%) [50].

Detecting and diagnosing gastric neoplasm with endoscopic findings is not a simple feasible process owing to difficulty of distinguishing its subtle typical feature change from gastric mucosal background and generalized inflammation [17,18,19]. It depends on the subjective decision of the endoscopist, especially regarding performing biopsies and making endoscopic diagnosis and assessments [24,25].

Considering high worldwide prevalence and importance of making early diagnosis for improvement in prognosis, various diagnostic methods, including endoscopic devices as well as molecular biomarkers have been approached and applied in the endoscopic clinical field. First, the application of endoscopic ultrasonography (EUS) has beneficial diagnostic capability for submucosal masses compared with conventional endoscopy as well as CT. It is able to distinguish and characterize submucosal lesions, such as leiomyoma, gastrointestinal stromal tumor (GIST), and gastric duplication cyst (GDC) [56]. Since no definite exclusion of malignancy potential is present, application of the EUS method would result in both earlier diagnosis with improvement in prognosis. Second, molecular biomarkers offer a non-invasive method to detect early-stage gastric cancer with mi-RNAs from gastric juice or other fluids are gaining growing focus [57]. Considering its role of regulating gene expression and tissue specificity, micro-RNA molecules serve as high potential tool resulting in aiding earlier identification of gastric cancer.

Diverse optical development enhancing endoscopic visual fields and assisting endoscopic detection rate of early malignant lesion features has been introduced, which include narrow-band imaging (NBI) and magnification endoscopy in combination with WLE [20]. However, various endoscopic modalities in making endoscopic assessments inevitably depend highly on the levels and clinical training experience of endoscopists, which supports the presence of missing diagnosis and interval cancer [22,23].

In contrast, currently developed and introduced AI algorithm models are free from those issues mentioned above regarding the high dependence of endoscopist subjective decision and endoscopic experience.

As the introduction of artificial intelligence is becoming common and generalized in the medical field, broad application is performed in the endoscopic field from the detection and classification of neoplasm to the prediction of invasion of depth. Several previous studies that have made attempts to detect gastric neoplasms are presented as follows: Hirasawa et al. [43] applied a CNN-based algorithm called the Single Shot MultiBox Detector for the detection of early gastric cancer and it has presented a result of sensitivity of 92.2% and PPV of 30.6%. Sakai et al. [58] trained a CNN-based system with an outcome of 82.8% accuracy in detecting gastric cancer. In addition, Li et al. [45] used magnified NBI images of EGC for training the Inception-v3 CNN model with results of sensitivity of 91.2%, specificity of 90.6%, and accuracy of 90.9%. Other studies focusing on the classification of gastric lesions are summarized as follows: Lee et al. [44] make use of the Inception-v3 network, ResNet50, and the Visual Geometry Group (VGG) Net to differentiate gastric ulcers and neoplasm with a diagnostic accuracy of 0.9649 for differentiating between normal vs. cancer, 0.9262 for differentiating between normal vs. ulcers, and 0.7712 for differentiating between cancer vs. ulcers. Cho et al. [59] have developed a deep learning model with three CNN architectures of Inception-v4, ResNet-152, and Inception-ResNet-v2 for training and validating the classification of conventional endoscopic images. The outcome of the model has come out with an accuracy of 84.6% (95% CI: 83.69–85.5). Finally, several studies making advancements on expecting depth of lesions are listed next. Zhu et al. [60] validated ResNet50 for the prediction of invasion depth with an overall accuracy of 77.5% of the experienced endoscopists and 76.5% sensitivity, 95.6% specificity, 89.7% PPV, and 89.0% NPV. Yoon et al. [61,62] trained and validated the lesion-based VGG16-network and gradient-weighted class activation mapping (Grad-CAM) for EGC detection and invasion depth prediction with an overall AUC for EGC detection and invasion depth prediction of 0.981. Cho et al. [63] used Inception-ResNet-v2 and DenseNet-161 models to evaluate the invasion of the depth of gastric neoplasm. The AUC and diagnostic accuracy for determining the invasion depth were 0.887 and 77.3%, respectively.

As presented in our study result ALPHAON^®^ has been proven to improve the performance of beginner endoscopists in making significant progress and enhancement in accuracy 0.94 (0.93–0.96, *p* < 0.001), sensitivity 0.87 (0.82–0.92, *p* < 0.001) and specificity 0.96 (0.95–0.97, *p* < 0.001). As with other recent multicenter diagnostic studies [49,50], this has presented the possibility of ALPHAON^®^ performing as an endoscopic educator.

Therefore considering notable improvement outcomes in beginner endoscopists, ALPHAON^®^ has demonstrated the potential to serve as an endoscopic educator for beginners, monitoring tutors in the practical field, reducing clinical load and endoscopic guidance in developing countries in need of endoscopic specialists.

By embedding expert skills in a real-time algorithm as encoding, AI automatically transfers high clinical endoscopic knowledge and skills of experts to other clinicians contributing to the equalization of the gastroenterology community. With the assistance of AI, human–machine interaction does not only depend on the performance of individual physicians, but allows for overcoming the weak points of endoscopists such as miss rate or localization of upper GI neoplasia.

Even with unchanging consistency, the sole performance of the AI system and its detection with augmentation applied to the endoscopic field does not ensure diagnostic value without the decision of a clinician. Technical environments of AI are considered low-invasive devices expected to assist and support but not substitute clinical endoscopists. Therefore, interaction between AI machines and physicians is necessary for reasonable diagnostic assessment and determination. The gastric mucosal lesion that is detected as an abnormal finding could be evaluated as a false positive by endoscopists. On the other hand, lesions missed by a machine or physician could be supported and made complementation by human–machine interaction owing to the physician’s distraction and lack of expertise. But there still are limitations of false positive detection that result in tired distraction of endoscopists and an unnecessary resection. False positive results in a weighted burden on medical financial costs and additional biopsies causing potential over or mismanagement during endoscopic exams.

In the aspect of presenting significant advancement and progression in specificity and sensitivity after application, though limited in beginners, ALPHAON^®^ has shown the potential to reduce false positives and missing rates of early gastric neoplasm. Frequent false positive findings of CADe result in fatigue of operators and low cost-effectiveness of endoscopic exams raising concerns about its efficacy. This issue is evident, especially for endoscopists on training courses in lack of discriminating true gastric abnormal lesions with a false positive result of CADe. Therefore, enhancing sensitivity and reducing the false positive ratio with optimization of algorithm and database would result in maximization of true benefits and promising outcome of CADe. As the main false positive factors are normal gastric structures including esophageal junction, mucus, and pyloric ring, improvement regarding false positives would result in reducing fatigue of recognizing normal components with clinical diagnostic outcome. In addition, the application of ALPHAON^®^ with higher sensitivity in beginners would lead to better treatment results of early cancerous lesions diagnosed in the early stage with endoscopic management.

Several previous studies on detecting and diagnosing gastric lesions have shown promising results of AI performing as supportive assistance [43,44,45,46,47]. Also, comparison with the endoscopic level of experts and trainees in evaluating and validation of AI model has been reported in prior studies [49,50]. In our study, grouping and classifying endoscopists in three levels of clinical endoscopic experience from multi-center has given compatible features and supporting evidence with the advantage of being performed in an area of one of the world’s highest prevalence of gastric cancer.

Besides these favorable outcomes, there are some concerning limitations taken in to consideration on ALPHAON^®^.

First, apart from actual current real clinical field endoscopic environment similar with real-time video clips or images including NBI or magnification image, datasets were prepared and tested with white-light still cut images.

Second, possibility of selection bias would be considered regarding retrospectively obtained datasets in the study, as well as the limitation of reflecting current real medical application.

Third, being a CADe system, ALPHAON^®^ has further to be trained as computer-aided diagnosis (CADx) algorithm with more clinical information including pathologic result with endoscopic data for giving more advanced practical clinical diagnostic evaluation.

Finally, by using datasets from the same center for training and validation, this study could not be excluded from having overfitting bias.

Regarding the limitations of our study discussed above, following future perspectives would make further advancement and give enhancement in the upcoming artificial intelligence era of the endoscopic field.

Following prospective studies in multicenter would give supportive validation result of CADe algorithm system with reducing and overcoming overfitting bias. Also, advancement made to computer-aided diagnosis (CADx) algorithm with more clinical information including pathologic result with endoscopic data for giving more progressed practical clinical diagnostic evaluation.

## 5. Conclusions

In this study, the high validation performance state of CADe algorithm system, ALPHAON^®^, detecting gastric neoplasm in upper endoscopy was verified. Also, CADe algorithm system has demonstrated its potential to serve as endoscopic educator for beginner improving and making progression in sensitivity and specificity resulting in detecting better outcome of early gastric neoplasm. It is critical to recognize the limitation of AI being an absolute substitute for human clinician in current medical field and technical status, but it is important not to underestimate its beneficial role of being supportive assistance and practical guidance. Prospective validation in multicenter studies would be required before application of CADe in clinical field for delicate endoscopic screening.

## Figures and Tables

**Table 1:** Comparison between ALPHAON^®^ and endoscopists on validity in first and second study.

	Accuracy (95% CI)	Sensitivity (95% CI)	Specificity (95% CI)
ALPHAON	0.88 (0.85 to 0.91)	0.93 (0.88 to 0.98)	0.87 (0.84 to 0.90)
Experts	0.99 (0.98 to 0.99)	0.97 (0.94 to 0.99)	0.99 (0.99 to 1.00)
Trainees	0.96 (0.95 to 0.97)	0.85 (0.81 to 0.89)	0.99 (0.98 to 0.99)
Beginners	0.88 (0.86 to 0.90)	0.73 (0.67 to 0.79)	0.92 (0.90 to 0.93)
ALPHAON + experts	0.97 (0.97 to 0.98)	0.91 (0.87 to 0.95)	0.99 (0.99 to 1.00)
ALPHAON + trainees	0.97 (0.96 to 0.98)	0.88 (0.83 to 0.92)	0.99 (0.99 to 1.00)
ALPHAON + beginners	0.94 (0.93 to 0.96)	0.87 (0.82 to 0.92)	0.96 (0.95 to 0.97)

## Data Availability

The original contributions presented in this study are included in the article. Further inquiries can be directed to the corresponding author.
